# Mallet Finger Lattice Casts Using 3D Printing

**DOI:** 10.1155/2019/4765043

**Published:** 2019-07-01

**Authors:** Hyeunwoo Choi, Anna Seo, Jongmin Lee

**Affiliations:** ^1^Department of Biomedical Engineering, Kyungpook National University, Daegu 41944, Republic of Korea; ^2^Lee Gil Ya Cancer and Diabetes Institute, Gachon University of Medicine and Science, Yeonsu-gu, Incheon, Republic of Korea; ^3^Department of Radiology, School of Medicine, Kyungpook National University, Daegu 41944, Republic of Korea

## Abstract

Currently, research based on the technology and applications of 3D printing is being actively pursued. 3D printing technology, also called additive manufacturing, is widely and increasingly used in the medical field. This study produced custom casts for the treatment of mallet finger using plaster of Paris, which was traditionally used in clinical practice, and 3D printing technology, and evaluated their advantages and disadvantages for patients by conducting a wearability assessment. Mallet finger casts produced using plaster of Paris, when incorrectly made, can result in skin necrosis and other problems for patients. These problems can be mitigated, however, by creating casts using 3D printing technology. Additionally, plaster casts or ready-made alternatives can be inconvenient with respect to rapid treatment of patients. In contrast, 3D-printed casts appear to provide patients with appropriate treatment and increase their satisfaction because they are small in size, custom-made for each patient, and can be quickly made and immediately applied in clinical practice.

## 1. Introduction

Mallet finger refers to a deformity in which the finger cannot be extended due to severing of the extensor tendon at the distal phalanx or fracture of the bone to which it attaches. Mallet finger can be classified as tendinous, in which only the tendon is ruptured, or bony, in which it is accompanied by a broken phalanx. Surgical intervention is required in the case of bony mallet finger. However, tendinous mallet finger is treated conservatively, primarily by using a splint to extend the finger so that the tendon reattaches naturally. Traditional conservative treatments include fixation of plaster of Paris or metal casts, whereas surgical interventions include bone fragment fixation using steel wire, fixation with lag screws, tension band wire fixation, fixation using figure-eight tension band wire, intramedullary wire fixation, and dorsal suture.

Three-dimensional (3D) printing technology is widely used in industry, biology, and medicine [1]. Major applications of the technology include prosthetic fingers made using 3D scanners and printers [2], wrist protection devices [3], and synthetic bone implants made of calcium phosphate scaffolding using low-temperature 3D printing technology [4]. Custom-made orthopaedic wrist casts can be created using techniques such as fused deposition modelling with rapid prototyping (FDM-RP) and 3D printing [5–8]. 3D printing technology offers significant advantages in biomedical instrumentation and tissue engineering because of its ability to produce small quantities or single parts when needed based on patient-specific requirements [9] owing to its ability to produce recognizable 3D objects applied to surgery planning, orthoses, and related application programs [10], as well as the capability to produce single or small quantities of parts according to patient-specific needs.

Medical applications of 3D printing are rapidly expanding, and revolutions in medical services are expected [11]. The present study attempts to use a 3D printer to produce lattice casts that provide good ventilation, hygienic treatment, and clear X-ray images for treatment of mallet finger.

## 2. Materials and Methods

### 2.1. Subjects

Subjects of the study were selected randomly. Casts were designed for subjects' index fingers using either the traditional plaster of Paris method or MediACE 3D software (based on 3D scanning to measure the finger's dimensions).

### 2.2. Production of Plaster of Paris Casts

**Figure 1:**
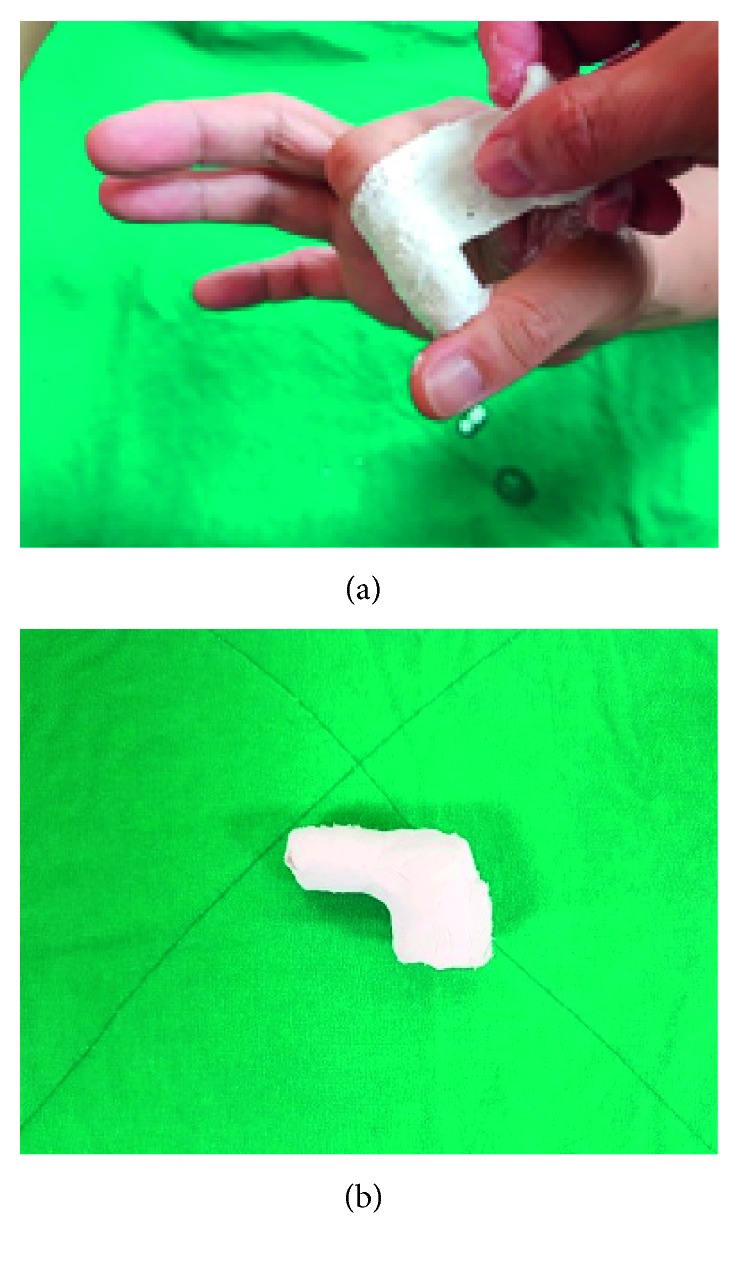
Plaster of Paris casts traditionally used to treat mallet finger in clinical practice. (a) Procedure for creating cast. (b) Removed plaster of Paris cast.

Plaster of Paris casts, which are traditionally used in clinical practice to treat mallet finger, were created as shown in Figure 1(a).  Figure 1(b) shows a removed plaster of Paris cast.

### 2.3. Production of 3D-Printed Casts

**Figure 2:**
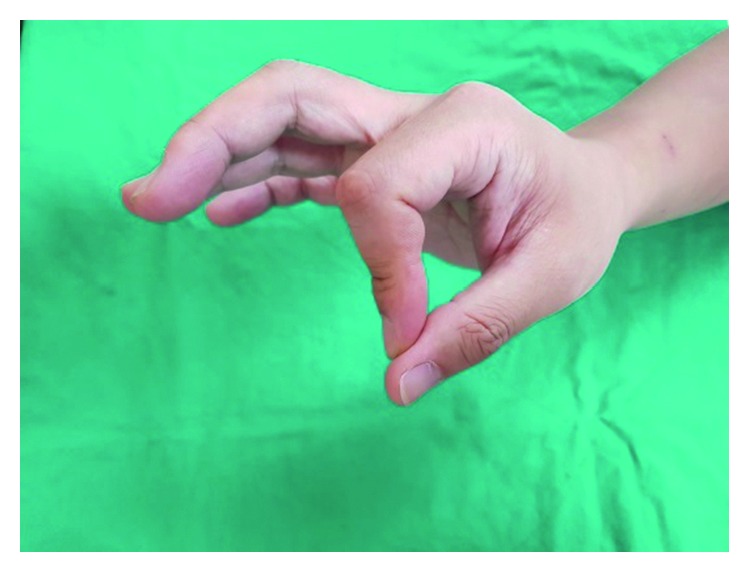
Finger maintaining posture of the extensor tendon.

First, the index finger was scanned repeatedly while maintaining posture of the extensor tendon (Figure 2) using a 3D scanner (3D Systems Sense scanner) to generate a stereolithography (STL) file of the finger. STL files, the end result of the 3D modelling process, are typically generated by computer-aided design (CAD) programs, which use this file type to store information about 3D models. The STL file shows the surface shape of a 3D object without representation of colour, texture, or other general characteristics of the model. Accordingly, STL files are widely used for rapid prototyping, 3D printing, and computer-aided manufacturing.

**Figure 3:**
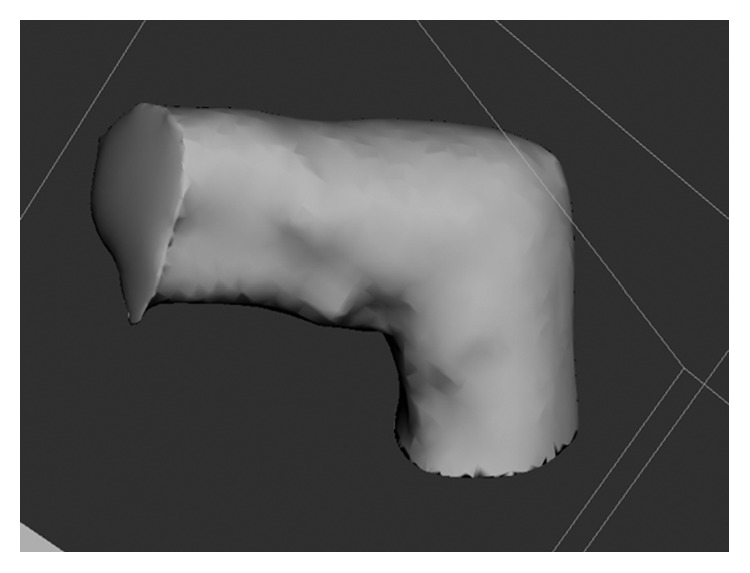
STL file of the mallet finger generated by the 3D systems sense scanner.

**Figure 4:**
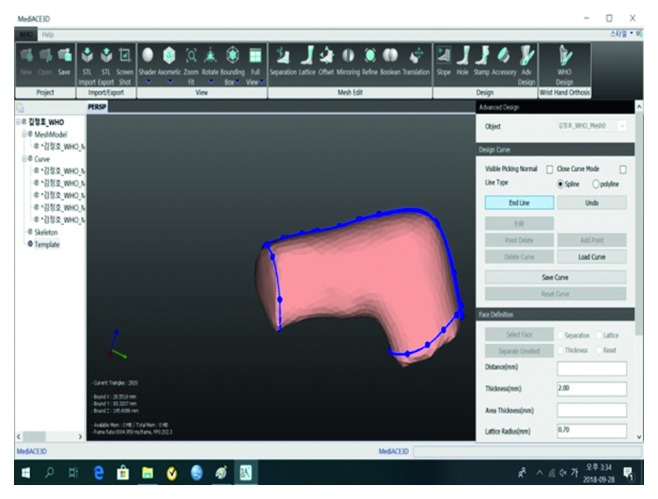
MediACE 3D masking model.

**Figure 5:**
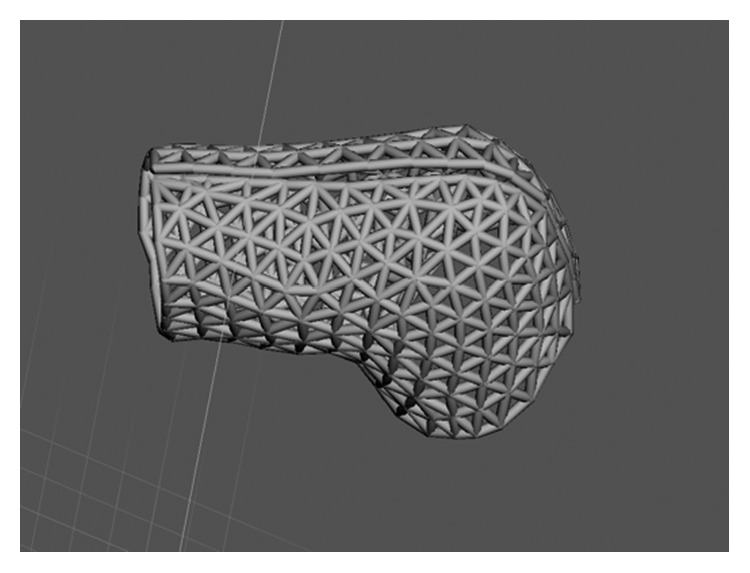
Final lattice design of 3D-printed mallet finger cast.

A 3D model of the mallet finger cast was then created based on the STL file (Figure 3) using the MediACE 3D software (Figure 4), and the final lattice design of the 3D-printed cast was completed (Figure 5).

The MediACE 3D software is expected to create beneficial business value in the medical industry and to contribute to implementation, distribution, and diffusion of customised medical services using 3D printing, the foundation of the 4th Industrial Revolution. It has been verified through clinical application and evaluation.

**Figure 6:**
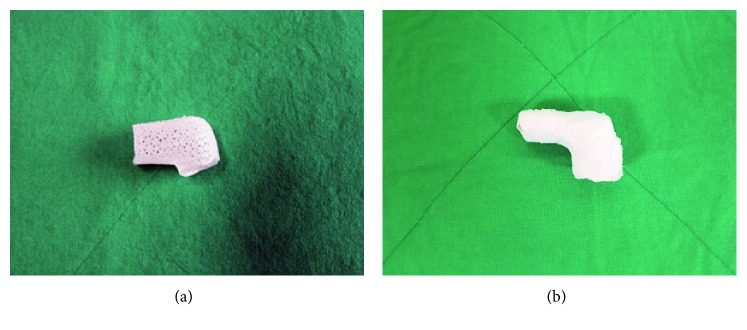
Comparison of removed mallet finger casts. (a) 3D-printed lattice cast. (b) Plaster of Paris cast.

A mallet finger cast for 3D printing was designed using the mallet finger STL file and MediACE 3D and then converted into G-code. The cast, shown in Figure 6(a), was produced using polylactide (PLA) resin on a 3D Edison printer. For comparison, a plaster of Paris cast is shown in Figure 6(b).

### 2.4. Comparative Evaluation of Products

**Figure 7:**
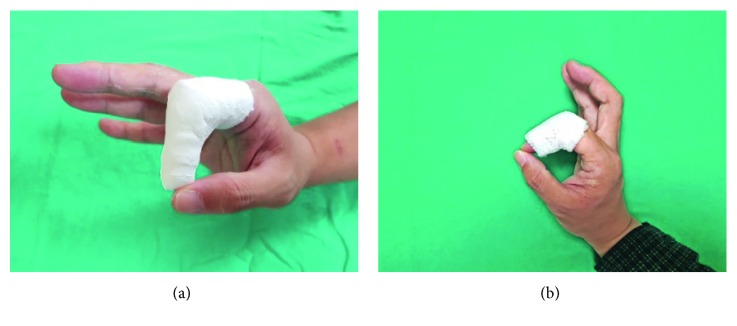
Application of mallet finger casts. (a) Plaster of Paris cast. (b) 3D-printed lattice cast.

Application of the plaster of Paris and 3D-printed casts is shown in Figure 7. To evaluate the performance of and patients' satisfaction with each type of cast, a satisfaction evaluation (Quebec User Evaluation of Satisfaction; QUEST) [12] and a wearability evaluation (Product Performance Program; PPP) were conducted. The items of both measures were rated on a 5-point Likert scale from 1 (negative end of scale) to 5 (positive end of scale).

## 3. Results and Discussion

3D-printed lattice casts for treatment of mallet finger were produced based on STL files obtained through 3D scanning, the MediACE 3D software, and 3D printing technology. The advantages and disadvantages of the 3D-printed casts were then compared with those of plaster of Paris casts traditionally used in clinical practice.

The 3D-printed lattice casts showed results similar to those of the traditional plaster of Paris casts in the QUEST evaluation (Table 1). However, overall, subjects indicated that they were “very satisfied” or “satisfied” with the 3D-printed casts' dimensions, weight, adjustment, ease of use, and comfort. Subjects were more satisfied with the weight and ease of use of the 3D-printed casts than the plaster of Paris casts (Table 1). Overall, the 3D-printed casts received a rating of “very satisfied” in the PPP wearability assessment (Table 2). In contrast, the plaster of Paris casts received many negative or “not applicable” ratings because they must be applied by medical staff.

The lattice design of the 3D-printed casts and their customised application can prevent necrosis or infection, offer a thickness and elasticity that guards against slipping off, and resolve other shortcomings of traditional plaster of Paris casts, including oedema, discoloration, hinderance of function and circulation, pain, pulselessness, dysesthesia, and burning from pressure. In future, 3D-printed lattice casts should be used instead of plaster casts to treat mallet finger, based on their many benefits, such as providing patients with the most appropriate, customised treatment.

## 4. Conclusions

This study produced 3D-printed lattice casts for treatment of tendinous mallet finger based on STL models of the index fingers of randomly selected subjects, obtained using a 3D Systems Sense scanner. The casts were constructed from PLA resin using a 3D Edison Printer.

The advantages of traditional plaster of Paris casts are the difficulty of taking it off by patients once it is worn and short application time. Disadvantages include possible pain, skin oedema, discoloration, burning, infection, and necrosis; the complications of splint treatments are mainly skin-related.

Complications occurring in 123 cases of mallet finger treated both surgically and nonsurgically were reviewed. The 84 cases of splint treatment had a complication ratio of 45%; these complications were almost always temporary. Among the 45 cases of surgical treatment, 53% experienced complications and 76% of complications developed during the average monitoring period of 38 months. Six cases that involved surgical treatment after splinting (for a minimum of six weeks) were counted in both groups. Major complications experienced by surgically treated patients were deep infection (4%), total joint incongruity (18%), and nail deformity (18%). Seven patients (16%) required reoperation, and all had unsatisfactory results [13] except for one whose result was unknown.

The 3D-printed lattice cases were compared with plaster of Paris casts in treatment of patients with mallet finger. The results of PPP evaluation indicated that 3D-printed lattice casts allow for accurate, rapid production of customised orthoses, improving upon existing production methods. The 3D-printed casts can additionally resolve problems associated with plaster of Paris casts, namely, hyperextension and skin problems, because production is customised to the patient using 3D scanning. The size is very appropriate for 3D printer output, and it uses less material than a plaster of Paris cast and requires little output time. The ability of 3D-printed casts to overcome the disadvantages of traditional plaster of Paris casts makes them beneficial for clinical application.

Development of 3D-printed lattice casts is meaningful because they have the potential to help many patients if produced in clinical practice using 3D printers. Improvements in body scanning technology and product design software are expected to bring benefits to clinical practice. Future research should investigate the production of orthoses with various structures using the processes developed in this study. The investigators hope that the present study may serve as a reference for research on the production methods of finger orthoses based on various diseases and their treatment in clinical practice, such as the various types of 3D-printed finger casts in Figure S1.

## Figures and Tables

**Table 1:** User satisfaction evaluation of 3D-printed and plaster of Paris casts for mallet finger (QUEST).

Item	3D-printed lattice casts		Plaster of Paris casts	
	Score	Details	Score	Details
1. Dimensions	5		3	Determined by practitioner
2. Weight	5		3	Heavy
3. Adjustment	5		3	Difficult to adjust once hardened
4. Safety	5		3	Skin necrosis possible
5. Durability	5		3	Contamination possible
6. Ease of use	5		5	Must be applied by practitioner
7. Comfort	4		3	Determined by practitioner
8. Effectiveness	4		4	Determined by practitioner
Total score	4.75		3.5	

*Note.* The three most important items, in descending order, were dimensions, weight, and safety.

**Table 2:** Wearability evaluation of 3D-printed and plaster of Paris casts for mallet finger (PPP).

Questionnaire items		3D-printed	Plaster of Paris
No.	Item		
1	I like to use it	5	4
2	It can be used without complicated action or operation	5	0
3	It can be used anywhere and anytime	5	0
4	The procedure for wearing it is simple and uncomplicated	5	0
5	I can understand the principle of the cast	5	5
6	There is no danger or risk of malfunction	5	0
7	Wearing it incorrectly will not cause damage	3	0
8	It is comfortable to wear	5	0
9	Little effort is required to wear it	5	0
10	There are no requirements when using it	5	0
11	It is easy to use the fingers while wearing it	5	5
12	It is an adequate size and shape to protect the fingers	5	3
13	The size and shape are suitable for carrying or keeping it	5	0
14	The colour and shape are good	4	2
15	It is easy to clean and care for	5	2
16	It does not cause skin problems	5	3
17	The cast strength is good	5	5
Total score		4.82	1.70

*Note.* Scoring: 5 = strongly agree; 4 = agree; 3 = neither agree nor disagree; 2 = disagree; 1 = strongly disagree; 0 = not applicable.

## Data Availability

The data used for evaluation purposes are uploaded and made publicly available.

## Supplementary Materials

Supplementary Materials Figure S1: various types of 3D-printed finger casts.
