# Supplementary figures and images for: Mallet Finger Lattice Casts Using 3D Printing
Source: J Healthc Eng. 2019 Jul 1;2019:4765043. doi: 10.1155/2019/4765043 (PMC6636505; doi:10.1155/2019/4765043)

## Supplementary Materials

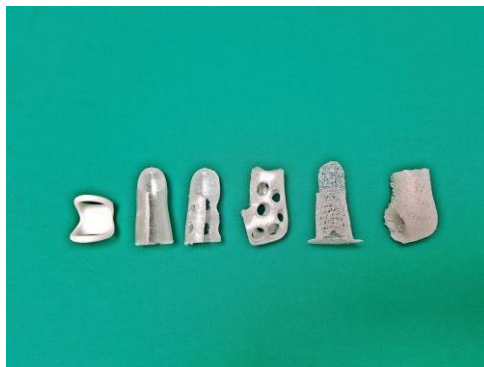

Figure S1: Various types of 3D-printed finger casts.

Supplement: Supplementary Materials — Figure S1: various types of 3D-printed finger casts. [file 4765043.f1.pdf]
